# PEO Treatment for Improved Corrosion Resistance in a Zn-Mg Alloy: Electrochemical and Structural Analysis

**DOI:** 10.3390/ma18174064

**Published:** 2025-08-29

**Authors:** Ramona Cimpoeșu, Sorin Georgian Moga, Bogdan Istrate, Fabian Cezar Lupu, Nicanor Cimpoesu, Ana-Maria Roman, Gheorghe Bădărău, Ion Pătrașcu, Remus Diaconu, Romeu Chelariu

**Affiliations:** 1 Department of Materials Science, Faculty of Materials Science and Engineering, “Gheorghe Asachi” Technical University of Iasi, 41 Dimitrie Mangeron Blvd., 700050 Iasi, Romania; ramona.cimpoesu@academic.tuiasi.ro (R.C.); nicanor.cimpoesu@academic.tuiasi.ro (N.C.); ana-maria.roman@academic.tuiasi.ro (A.-M.R.); gheorghe.badarau@academic.tuiasi.ro (G.B.); 2 Regional Center of Research and Development for Materials, Processes, and Innovative Products Dedicated to the Automotive Industry, National University of Science and Technology Politehnica Bucharest, Pitesti University Centre, Targu din Vale, No. 1, 110040 Pitesti, Romania; sorin_georgian.moga@upb.ro; 3 Faculty of Mechanical Engineering, “Gheorghe Asachi” Technical University of Iasi, 43 Dimitrie Mangeron Blvd., 700050 Iasi, Romania; bogdan.istrate@academic.tuiasi.ro (B.I.); fabian-cezar.lupu@academic.tuiasi.ro (F.C.L.); 4 SC iP AUTOMATIC DESIGN SRL, 6 Industriei Str., 117435 Lunca Corbului, Romania; patrascu@ipad.ro (I.P.); dremus@ipad.ro (R.D.)

**Keywords:** ZnMg alloy, plasma electrolytic oxidation, corrosion resistance

## Abstract

Zinc-based alloys have been extensively studied for their potential applications in biodegradable materials, yet their corrosion behaviour necessitates the development of effective surface treatments. In this study, a ZnMg alloy was developed by casting in an inert medium and subsequently treating it with Plasma Electrolytic Oxidation (PEO). The corrosion behaviour was characterised in a 0.9% NaCl solution through Tafel polarisation, cyclic polarisation, and electrochemical impedance spectroscopy (EIS). Additionally, the surface morphology was investigated using scanning electron microscopy (SEM) and EDX analysis. The structure and phases of the oxide layer and of the corrosion products were investigated through X-ray diffraction (XRD). The electrochemical results demonstrated a substantial decrease in the corrosion current density and an increase in the polarisation resistance for the treated samples. Electrical Impedance Spectroscopy (EIS) modelling revealed the formation of a layer exhibiting distinct capacitive behaviour, comprising two distinct regions. XRD analysis confirmed evidence of corrosion compounds characteristic of chlorinated media on the surface. The findings indicated that PEO treatment enhanced the corrosion resistance of the ZnMg alloy, suggesting its suitability for biomedical applications or exposure to marine environments characterised by high levels of corrosion.

## 1. Introduction

Biodegradable zinc alloys have emerged as a promising alternative to traditional metal implants in the medical field. Zinc is present in small amounts in the body and plays an important role in bone development and cell growth processes [1,2]. Unlike many other metals used in surgical implants, zinc generates degradation products with minimal toxic effects [3]. Additionally, the rate of zinc degradation can be well controlled, ensuring progressive integration of the implant without causing excessive hydrogen production or sudden pH changes, which are common in other biodegradable materials, such as magnesium [4,5,6].

Many studies published in scientific journals confirm that Zn-Mg alloys exhibit superior mechanical properties and controlled degradation behaviour [7,8,9,10,11,12], which recommend them for use in biodegradable medical implants [13,14,15]. For example, Jin et al. [7] reported that the addition of Mg in small proportions (less than 0.1 wt.%) to Zn resulted in a significant increase in tensile strength, from 63 MPa for Zn-0.002Mg to 339 MPa for Zn-0.08Mg, and an increase in ductility [7]. These improvements were attributed to grain structure refinement and the formation of the intermetallic Mg_2_Zn_11_ phase, which contributes to the structural strengthening of the alloy.

Kubásek et al. [8] investigated the microstructure and in vitro degradation of Zn-Mg alloys with Mg content ranging from 0 to 1.6 wt.%. Their results showed the formation of intermetallic phases (Mg_2_Zn_11_ and MgZn_2_) distributed in the Zn matrix. The structure became finer with increased Mg content. The authors stated that the zinc alloys exhibited low corrosion rates. They concluded that the implanted devices would maintain 95% structural integrity for up to six weeks.

Gong et al. [9] conducted a study on the properties of biodegradable Zn-1 wt.%. Mg alloys in simulated body fluid (SBF). They reported that the corrosion rate of the extruded material was 0.12 ± 0.05 mm/year, which was slower than that of the cast material. The extruded material had a homogeneous microstructure, good mechanical strength, and biocompatibility.

However, a significant drawback associated with these materials is their high susceptibility to corrosion in chloride-rich environments. This can result in premature loss of mechanical integrity and uncontrolled release of degradation products.

In order to overcome these limitations, surface treatments have been thoroughly researched, and plasma electrolytic oxidation (PEO) has been shown to be a promising technology [16,17]. PEO facilitates the establishment of hard, adherent and porous oxide layers on the surface of metal alloys through dielectric-controlled discharges in the electrolyte at elevated voltages. It has been established that these layers can confer superior electrochemical protection and wear resistance, and they can be functionalised for bioactive applications [18].

Regarding Zn-based alloys, research on PEO remains relatively limited in comparison to that on Mg or Ti. A comprehensive characterisation of the influence of process parameters on the microstructure of the formed layer and the electrochemical performance is imperative [19,20]. While plasma electrolyte oxidation (PEO) treatment has been extensively studied in magnesium and titanium alloys, its application to zinc-based alloys, particularly ZnMg, remains limited, and the influence of process parameters on anticorrosive performance is insufficiently researched. Furthermore, the literature has not addressed in an integrated manner the detailed correlations between the structure of the oxide layer formed at different voltages, electrochemical behaviour phase composition (including corrosion products identified.

This paper makes an original contribution by investigating PEO-treated samples at variable voltages, focusing on evaluating the protective capacity of the formed layers and identifying specific corrosion compounds. Thus, the proposed study provides an in-depth understanding of the protective mechanisms offered by PEO treatment when applied to ZnMg, thereby supporting the potential of these alloys for use in corrosive environments, including in the biomedical field. The results revealed the formation of a complex oxide layer on the alloy’s surface, which functions as a protective barrier against corrosion in a saline electrolyte solution.

## 2. Materials and Methods

The development of the ZnMg with 3 wt.% Mg, alloy was accomplished within a laboratory furnace, utilising an Argon-controlled atmosphere and resistance heating techniques. A ceramic crucible and high-purity materials (Zn: 99.995% and Mg: 99.9%) were subjected to a preliminary step of oxide removal prior to elaboration. The casting process was conducted in a metallic mould that had been preheated to 300 °C. The molten metal bath was composed of zinc, which was heated to 650 °C. Magnesium was introduced beneath the zinc load initially.

For the oxidation process, an industrial equipment, SC iP AUTOMATIC DESIGN SRL, with a commercial 50 kW DC source (Plasma Technology Ltd., Hong Kong, China) with Constant Voltage, Constant Current, Constant Power working modes and bipolar, unipolar positive pulse and unipolar negative pulse output capabilities.

ZnMg alloy samples were machined in a cylinder shape with a 200 mm length and 6 mm diameter. Prior to the experiments, the samples were cleaned using ultrasonic treatment in acetone and subsequently dried with compressed air.

The experiments were carried out within a thermostatted glass reaction vessel cooled by a water chiller. The electrolyte volume was 1 L and consisted of 4 g/L KOH and 10 g/L Na_3_PO_4_ dissolved in distilled water. The conductivity and the pH of the electrolyte were 24.1 mS/cm and, respectively, 11.2. The cylinder-shaped samples served as anode, and a 316 L (low carbon) stainless steel sheet was used as cathode.

The samples were treated by the PEO process, in pulsed unipolar—potentiostatic mode. The samples used to analyse the structural and functional changes in the PEO coatings were prepared as follows: the process began with a 30 s passivation step at a constant voltage of 150 V [21,22], followed by a 150 s PEO treatment conducted at constant voltages of 250, 300, 350, and 400 V. The total process time for all the samples was 180 s. The sample code and the electrical regime are summarised in Table 1.

Linear polarisation, cyclic polarisation and electrochemical impedance spectroscopy (EIS) tests were performed to characterise the electrochemical behaviour of PEO-treated ZnMg alloy. The experiments were conducted within a three-electrode electrochemical cell, employing the oxidised sample as the working electrode with an exposed area of 0.26 cm^2^, a saturated calomel reference electrode, and an auxiliary platinum electrode. The measurements were performed in a 0.9% NaCl solution with an OrigaFlex0.5 potentiostat-galvanostat (OrigaLys ElectroChem SAS, Rillieux la Pape, France). Linear polarisation was recorded within the range of ±150 mV relative to the open circuit potential (OCP), with a scan rate of 0.5 mV/s. Cyclic polarisation was conducted between −1300 mV and +700 mV, with a scan rate of 5 mV/s. The electrochemical impedance spectroscopy (EIS) procedure was performed by applying a sinusoidal signal with an amplitude of 10 mV in the frequency range between 100 kHz and 10 mHz.

The investigation into the alloy surfaces post-oxidation and corrosion resistance testing was conducted utilising a Quattro C—ThermoFisher Scanning Electron Microscope (Thermo Scientific Quattro C, Brno, Czech Republic). Cross-sections of PEO-treated samples were prepared by Electrical Discharge Machining (EDM) cutting using a low-speed precision equipment with a metallic wire. The chemical composition of the surfaces was ascertained through EDS analysis employing an EDAX detector (Thermo Scientific Quattro C, Brno, Czech Republic.), utilising the Automatic, Element List and Mapping analysis modes. The X’PERT PRO MRD (Panalytical, Almelo, The Netherlands) X-ray diffractometer utilises a Cu(Kα) anode.

## 3. Results

### 3.1. Analysis of Oxidation Layers

**Figure 1:**
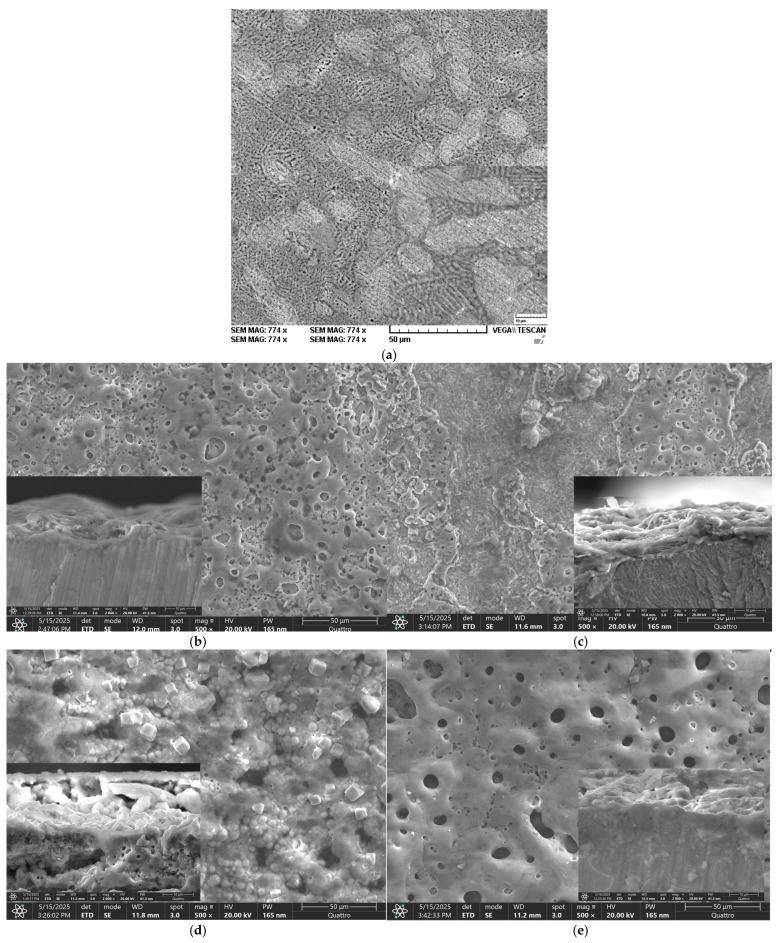
SEM images of sample surfaces, initial and after PEO and cross-section details (**a**) ZnMg-initial, (**b**) ZnMg-PEO250, (**c**) ZnMg-PEO300, (**d**) ZnMg-PEO350 and (**e**) ZnMg-PEO400.

The investigation of the oxidised samples was conducted through scanning electron microscopy. The analysis of the SEM images (see Figure 1) revealed that the ZnMg-PEO250 (250 V) sample exhibited a relatively smooth surface, low porosity, a few small round pores, and a few fine cracks. The surface appeared uniform, yet it was observed to be thin with moderate initial oxidation. It has been observed that weak discharges produce a fine and compact layer, but with limited protection. The ZnMg-PEO300 specimen (300 V) displays a conspicuously uneven surface texture, characterised by deeper and irregular pores, randomly dispersed thin cracks, and an augmented degree of textural non-uniformity. It has been shown that higher levels of tension are associated with increased occurrences of energetic discharges. A thicker layer has been observed to develop, accompanied by the emergence of internal stresses. This has led to the potential for local cracking and an increase in permeability.

With regard to the ZnMg-PEO350 (350 V) sample, the surface was found to be homogeneous, with evenly distributed round pores. The texture exhibited a high degree of density and regularity, with no visible cracks. A continuous and well-formed appearance has been observed. An optimal PEO regime has been identified. The coating is characterised by equilibrium in terms of thickness, porosity and adhesion. It is noteworthy that it offers optimal mechanical and corrosion protection. For the ZnMg-PEO400 (400 V) specimen, a highly uneven and unstable surface with large, deep, and irregularly arranged pores was observed.

There is evidence of multiple, extensive cracks between the porous areas. It is hypothesised that one cause of the issue is a high voltage, which has been shown to cause violent discharges. These, in turn, have been shown to lead to the formation of defects, exfoliation and loss of layer integrity. The layer was characterised by its substantial thickness, yet it exhibited a deficiency in cohesion.

Table 2 provides a detailed description of the surface chemical composition of the PEO1-ZnMg-PEO400 samples. Zn is the primary constituent of the alloy. It is notable that the presence of high content at 250–300 V is indicative of a thin layer, which consequently leaves the substrate detectable. At 350 V, Zn has been observed to decrease, indicative of an increase in oxide layer thickness and enhanced coverage. At 400 V, an increase is observed, which may indicate the onset of non-uniformity or cracking in the layer. At 350 V, a significant increase in oxygen is recorded, suggesting that oxide layer formation is at its peak at this point. At 300 V, a slight decrease is observed, suggesting reduced efficiency in the oxidation process. High values indicate the significant involvement of anodic oxidation in the alkaline solution. Na originates from Na_3_PO_4_ and is incorporated into the PEO layer. An increase in the voltage was observed, indicating that intense discharges facilitate Na^+^ diffusion in the layer. Sodium does not form part of the alloy, but it becomes a significant component of the oxide layer. The initial Mg content in the alloy was found to be 0.5 wt.%, with elevated values denoting hardened oxide layers. At a voltage of 350 V, the maximum is attained, resulting in the oxidation and concentration of Mg in the layer. The decline observed at 400 V indicates either volatilisation or preferential dissolution during periods of particularly intense discharge. Phosphorus is derived from the chemical compound Na_3_PO_4_. It is important to note that the minimum voltage required for the reduction of phosphate anions is 300 V. This process is most efficient at voltages between 350 and 400 V, which supports the hypothesis that higher voltages promote anionic incorporation. The element K originates from the chemical compound KOH. The maximum value observed was 350 V, which indicates that at this voltage, there is a higher tendency for K^+^ migration and incorporation. As indicated by the data, at 300 and 400 V values are low, which may be indicative of an insufficient layer or volatilisation at 400 V.

**Figure 2:**
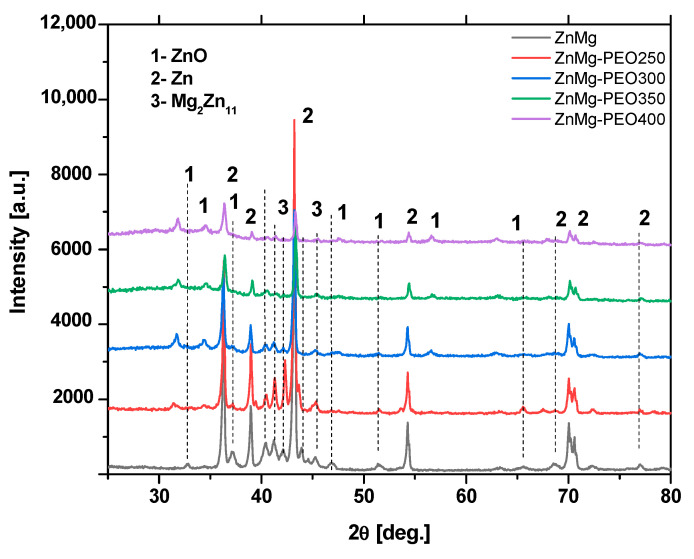
Diffractograms of an untreated ZnMg alloy and samples subjected to PEO at different applied voltages (250–400 V).

X-ray diffraction (XRD) analysis of the ZnMg samples subjected to PEO treatment (Figure 2) at varying voltages (250–400 V) has been shown to result in substantial alterations to the phase composition of the surface layer when compared with the untreated alloy. In regard to the untreated ZnMg sample, the presence of Zn metal phases (DB—04-005-9306) and Mg_2_Zn_11_ (DB 004-007-1412) intermetallic phases was predominantly observed. These phases are characteristic of the fundamental structure of the alloy. The application of PEO treatment resulted in the emergence of a new phase, designated “1” for ZnO (DB—04-015-0825), in all strains examined, suggesting the formation of a surface oxide layer.

### 3.2. Electro-Chemical Corrosion Resistance Analysis on Oxidised Layers

**Figure 3:**
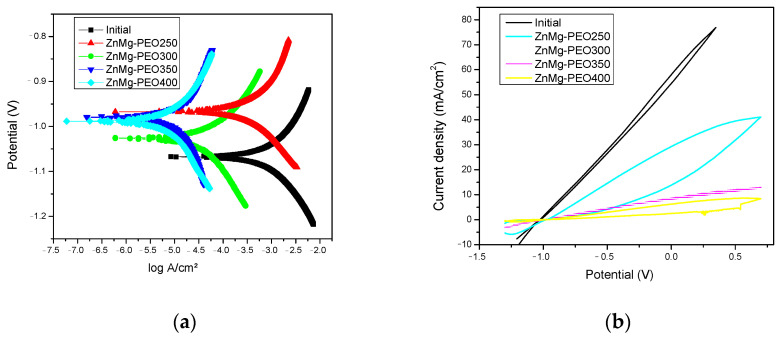
(**a**) Tafel curves and (**b**) cyclic polarisation curves for untreated and PEO-treated ZnMg alloy samples in 0.9% NaCl solution.

An investigation was conducted into the influence of PEO treatment on the electrochemical behaviour of a ZnMg alloy. To this end, a series of Tafel polarisation (Figure 3a) and the associated electrochemical parameters (Table 3), cyclic polarisation (Figure 3b) and electrochemical impedance spectroscopy (EIS) tests were performed in 0.9% NaCl solution using a three-electrode electrochemical cell.

Furthermore, the anodic and cathodic slopes for the oxidised samples differ significantly from the initial sample, suggesting a change in charge transfer mechanisms and slower kinetics of electrochemical reactions.

**Figure 4:**
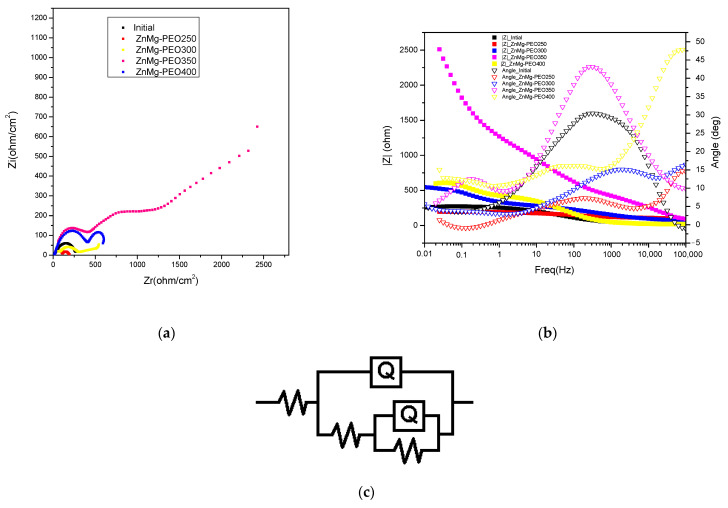
(**a**) Nyquist plots; (**b**) Bode plots for the untreated and PEO-treated ZnMg alloy (ZnMg-PEO250-ZnMg-PEO400) in 0.9% NaCl solution, and (**c**) the equivalent circuit used.

Electrochemical impedance spectroscopy (EIS) was performed to analyse the capacitive and resistive behaviour of the oxidised layers (Figure 4). Nyquist and Bode plots indicated a significant increase in the polarisation resistance (Rp) and a clear separation between two time constants, suggesting the formation of a double protective layer, comprising a porous outer and a compact inner layer.

The modelling of EIS data was performed using the equivalent circuit R(Q(R(QR))). The obtained parameters (see Table 4) confirmed the increase in charge transfer resistance and decrease in apparent electrical capacitance for the treated samples. This is indicative of superior protection against corrosion processes.

**Figure 5:**
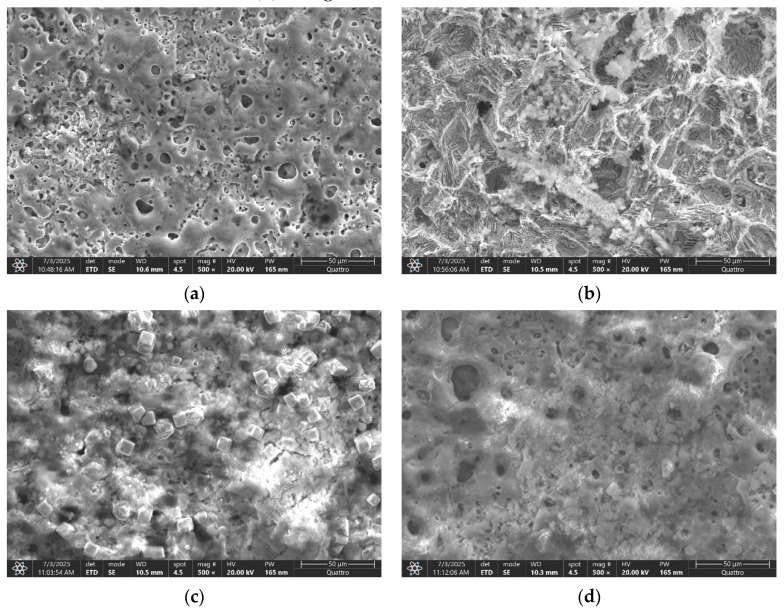
SEM images of sample surfaces after electro-chemical corrosion tests: (**a**) ZnMg-PEO250, (**b**) ZnMg-PEO300, (**c**) PEO3 and (**d**) ZnMg-PEO400.

As illustrated in Figure 5, SEM images of sample surfaces post-electrochemical corrosion tests are provided, including: (a) ZnMg-PEO250, (b) ZnMg-PEO300, (c) ZnMg-PEO350 and (d) ZnMg-PEO400.

Table 5 provides data on the surface chemical composition of the samples following electro-chemical corrosion resistance tests.

Prior to the corrosion process (see Supplementary File Figure S1), oxygen exhibited uniform and substantial distribution, suggesting the presence of a consistent oxide layer. Phosphates, derived from Na_3_PO_4_, were present and distributed uniformly, while Na and K were detected in the superficial, uniform regions. Concurrently, the presence of Zn was visibly diminished, indicating that the oxide layer covered the substrate efficiently. Mg, present in low percentages, exhibited minimal oxidation, but in trace amounts (according to the base alloy).

## 4. Discussion

A comparative analysis of the oxidised PEO layers in cross-section (see Figure S2 in the Supplementary File) revealed that for ZnMg-PEO250, a relatively thin yet compact, uniform and largely free of major visible discontinuities layer was obtained, with an estimated thickness of 2.5–4.0 μm (mean ≈ 3.2 μm). This outcome can be attributed to a low-intensity oxidation process, resulting in a uniform but limited-in-thickness layer. The ZnMg-PEO300 sample, which exhibited a thickness greater than that of the ZnMg-PEO250 sample, yet possessed a slight degree of porosity, was observed to contain micro-discontinuities and areas of uneven density. The estimated thickness of the ZnMg-PEO300 sample ranged from 5.0 to 7.0 μm, with a mean value of approximately 6.0 μm, as a result of an accelerated yet non-uniform oxidation process. In the case of the ZnMg-PEO350 sample, a dense, compact, continuous-textured layer with very good continuity was observed. No visible cracks or separations were identified, and the estimated average thickness was approximately 23.5 μm. This was attributed to an optimal oxidation process that formed a consistent, protective layer without any obvious exfoliation zones. The sample designated ZnMg-PEO400 exhibited a substantial layer characterised by microcracks and elevated porosity. The layer exhibited localised disruption, manifesting as vertical cracks, presumably resulting from the cutting process. The estimated thickness of the layer was found to be between 10 and 13 μm (with a mean of approximately 11.5 μm), a result of an intense oxidation process. However, this process also presented a risk of mechanical instability, as evidenced by the occurrence of cracks due to internal stresses.

It can be stated that, in the case of the ZnMg-PEO350 sample, the formed oxidised layer demonstrates a typical structure for successful PEO treatments, exhibiting a porous outer zone and a dense inner zone with good continuity and functional thickness [23,24]. The morphology of the material suggests that it is formed under controlled conditions, which would indicate that it has effective corrosion and wear protection properties.

The intensity of the diffractometric maxima corresponding to ZnO increased progressively with treatment voltage, reaching a maximum at 400 V. This suggests that the thickness and/or crystallinity of the oxide layer may be increasing.

Concurrently, the intensities of lines attributable to metallic Zn (2) and intermetallic phase-Mg_2_Zn_11_ exhibited a tendency to decrease, indicating an enhanced coating of the substrate or a potential partial transformation of these into oxide phases. The findings of this study demonstrate that PEO treatment results in the formation of an oxide-crystalline mixed layer, which possesses the inherent capacity to function as a corrosion inhibitor.

The Tafel curves (see Figure 3a) enabled the assessment of the corrosion potential (E_corr_) and the corrosion current density (i_corr_), which are pivotal parameters for the quantification of the corrosion rate. A shift of E_corr_ towards more positive values and a significant reduction of i_corr_ were observed for the PEO-treated samples compared to the initial sample, indicating improved electrochemical protection due to the formation of the oxide layer. The ZnMg-PEO350 sample exhibited the lowest corrosion current density, indicative of its superior electrochemical protection properties.

The parameters extracted from the Tafel curves (see Table 3) revealed a substantial enhancement in corrosion resistance following PEO treatment. The untreated sample exhibited a corrosion current density (j_corr_) of 0.277 mA/cm^2^ and a corrosion rate (v_corr_) of 3.87 mm/yr, associated with a reduced polarisation resistance (Rp) of 112 Ω·cm^2^. In contrast, the samples that were treated with PEO, specifically O3 and O4, exhibited a significant decrease in j_corr_, reaching 0.014 mA/cm^2^ and 0.032 mA/cm^2^, respectively. Concurrently, v_corr_ underwent a reduction to 0.199 and 0.451 mm/yr. This enhancement is substantiated by the elevated Rp values of 2210 and 1180 Ω·cm^2^ for O3 and O4, respectively, which are considerably higher than those of the original sample. In addition, a minor alteration in the corrosion potential (E_corr_) towards more positive values was detected for all PEO samples, indicating a more noble character of the oxide-dated surfaces.

The cyclic polarisation curves (see Figure 3b) provided additional information on the susceptibility to localised corrosion. The oxidised samples exhibited darker or absent hysteresis loops, suggesting a reduced tendency to oxidise in comparison to the untreated surface. The initial sample displays a substantially higher current density over the entire anodic range, reaching values exceeding 70 mA/cm^2^, indicating intense electrochemical activity and an increased susceptibility to generalised corrosion and possible pitting initiation. The ZnMg-PEO250 sample displays a moderately open hysteresis loop, which is indicative of the initiation of partial passivation mechanisms and a reduced, though not eliminated, susceptibility to localised corrosion. When considering the ZnMg-PEO300-ZnMg-PEO400 samples, the curves demonstrate a substantially diminished current density, thereby signifying the establishment of stable oxide layers with a high degree of efficiency in the inhibition of anodic reactions and the provision of effective protection against localised corrosion.

This evolution of cyclic behaviour suggests a progressive improvement in corrosion resistance with PEO treatment. The ZnMg-PEO400 sample exhibited the best electrochemical performance in terms of passivation and stability.

Nyquist plots (Figure 4a) show significant improvements in the electrochemical behaviour of the ZnMg alloy following PEO treatment. The untreated specimen displays a minor capacitive semicircle, suggesting a low polarisation resistance and, consequently, restricted corrosion protection. In turn, the samples treated with PEO demonstrate semicircles of increased diameter, attributable to elevated charge transfer resistance and the formation of protective oxide layers. The ZnMg-PEO350 sample displays the most substantial semicircle diameter, indicative of elevated polarisation resistance and an exceptional barrier capability against corrosive ions. The ZnMg-PEO300 and ZnMg-PEO400 samples also showed improvements over the initial state, though to a lesser extent, suggesting differences in the morphology, thickness or compactness of the oxide layer formed [25].

The existence of two time constants (as evidenced by the deviations from the ideal semicircles) is consistent with a porous layer-compact layer model and has been modelled using the circuit R(Q(R(QR))), as illustrated in Figure 4c.

Bode plots (see Figure 4b) furnish supplementary data concerning the electrochemical behaviour of the treated and untreated samples, thereby reflecting the efficacy of the oxide layers in acting as a barrier to corrosion processes. In the |Z| vs. frequency plot, it can be observed that the PEO3 sample exhibits the highest impedance values over almost the entire frequency range, with a maximum of over 2000 Ω at low frequencies (0.01–0.1 Hz). This indicates a significantly increased polarisation resistance and, therefore, a superior corrosion protection. In contrast, the ZnMg-PEO250 sample, and to a lesser extent the initial sample, exhibit considerably lower |Z| values, reflecting a poor efficiency of the formed layer. With regard to phase behaviour, ZnMg-PEO350 and ZnMg-PEO400 samples demonstrate high maximum phase angles (approximately 45°) across a broad frequency range (10–1000 Hz), suggesting a notable capacitive response linked to the development of a compact and stable oxide layer. The presence of a broad region where the phase angle remains high is indicative of extensive electrochemical passivity and the efficiency of the protective layer. The ZnMg-PEO250 samples, notably the untreated sample, demonstrate a low phase angle (below 20°) across the majority of the frequency range. This indicates a tendency towards a more resistive-like behaviour, consequently leading to a diminished pro-protection effect [26].

Therefore, PEO treatment leads to a significant improvement in the corrosion resistance [27] of the ZnMg alloy, attributable to the formation of stable, adherent and electrochemically passive oxide layers.

The equivalent circuit R(Q(R(QR)) has been utilised for the modelling of EIS data and has been found to be suitable for systems with two time constants, a characteristic of PEO-organic oxide coated materials. This circuit reflects the contributions of the electrolyte, the outer porous layer and the inner dense layer to the electrochemical response of the system. The primary parameters of the circuit (Table 4) were as follows: Rs (electrolyte resistance): This represents the solution resistance (NaCl 0.9%) between the working and reference electrode, Q1 (CPE—constant phase element, outer layer). This replaces an ideal capacitance in order to describe the nonlinear behaviour of the outer porous layer formed after PEO treatment. This element serves to reflect the heterogeneity and roughness of the surface, R1 (porous layer resistance), which is associated with the less compact portion of the oxide layer. An elevated value suggests a porous layer exhibiting enhanced resistance to ion penetration. Q2 (CPE, inner layer): This phenomenon is indicative of the capacitive behaviour exhibited by the dense, compact layer that is situated between the substrate and the porous layer. R2 (charge transfer resistance) is defined as the resistance to charge transfer at the metal/solution interface, which is influenced by the properties of the inner layer. An increase in this value is indicative of enhanced protection against the process of corrosion. The electrolyte resistance (Rs) exhibited relatively consistent values across the samples, ranging from 18 to 43 Ω·cm^2^, with no substantial impact on the interpretation. In contradistinction, the variations in the internal resistances and constant phase element (CPE) parameters provide a clear reflection of the efficacy of the applied treatment. The untreated specimen displays an exceptionally low charge transfer resistance (R1) of just 20 Ω·cm^2^, suggesting inadequate corrosion protection. The PEO treatment has been shown to induce a substantial increase in R1, particularly in samples ZnMg-PEO350 (1471 Ω·cm^2^) and ZnMg-PEO400 (419 Ω·cm^2^), thereby indicating the presence of an effective barrier at the metal-electrolyte interface. The low values of Q2 for ZnMg-PEO350 (4.66·10^−7^ Ss^n^/cm^2^) and ZnMg-PEO400 (5.5·10^−5^ Ss^n^/cm^2^) samples, which correlate with relatively constant n exponents (*n* ≈ 0.72–0.86), suggest an enhanced capacitive behaviour and the formation of more homogeneous and compact layers. The ZnMg-PEO400 sample exhibited an n2 exponent of 0.86, the closest to the ideal capacitive behaviour (*n* = 1), thus supporting the hypothesis of efficient electrochemical passivation. With regard to the outer porous layer, considerable variation is observed in Q1 and R1. The ZnMg-PEO350 sample is characterised by a high R1 (83 Ω·cm^2^), indicating a resistant porous layer, and a low Q1 value, suggesting a reduced porosity distribution and a more pronounced dielectric character.

The charge transfer resistance (R1), associated with the dense inner layer formed by microarc oxidation, reflects the ability of the electrochemical barrier to limit anodic processes. The untreated specimen displays an extremely low R1 value (20 Ω·cm^2^), signifying direct contact between the electrolyte and the metal substrate, lacking substantial protection. Subsequent to the implementation of PEO treatment, a progressive escalation of R1 was observed, with maximum values recorded for ZnMg-PEO350 (1471 Ω·cm^2^) and ZnMg-PEO400 (419 Ω·cm^2^). This finding suggests the formation of a compact and stable oxide layer, which is capable of effectively reducing charge transfer at the interface. This substantial increase in R1 is consistent with the capacitive behaviour observed in the Bode plots and with the large Nyquist half-circle diameters, thus confirming the superior effectiveness of the ZnMg-PEO350 treatment in improving corrosion resistance. The ZnMg-PEO300 sample, with an R1 of 309 Ω·cm^2^, indicates an intermediate level of protection. In contrast, ZnMg-PEO250 remains significantly below the others, with an R1 of only 71 Ω·cm^2^, suggesting the presence of a less dense or poorly coated layer. The corrosion protection efficiency (P.E.) was calculated based on the charge transfer resistance values obtained from EIS, using the method reported by Yang et al. [28]. The results showed a significant increase in P.E.% for the PEO-treated samples compared to the untreated ZnMg alloy: 71.8% (ZnMg-PEO250), 93.5% (ZnMg-PEO300), 98.6% (ZnMg-PEO350) and 95.2% (ZnMg-PEO400), respectively.

The strength of the porous layer (R2) provides relevant information on the continuity and efficiency of the outer barrier formed by microarc oxidation. The ZnMg-PEO350 sample shows an exceptionally high R2 value (1962 Ω·cm^2^), indicating a well-organised porous layer with low porosity and limited interconnectivity, which effectively prevents the penetration of aggressive ionic species. This value is almost 10 times higher than that of the untreated sample (232 Ω·cm^2^), suggesting extensive protection as early as the subsurface layer. The ZnMg-PEO300 sample demonstrates an intermediate value of 296 Ω·cm^2^, which is moderately higher than the initial sample. This finding suggests a partial improvement. In contrast, ZnMg-PEO250 (90 Ω·cm^2^) showed low R2 values, suggesting a more permeable pore structure or insufficient growth of the outer layer under these treatment conditions. Thus, ZnMg-PEO350 exhibits a clear synergy between the resistance of the porous layer (R2) and the inner layer (R1), both of which have high values, which explains the superior behaviour observed in the Nyquist and Bode plots.

Yao et al. [29] reported a corrosion current density of 8.787 × 10^−6^ A/cm^2^ and an E_corr_ of −1.01 V vs. SCE for a Zn-3Mg alloy in 3.5% NaCl. In our study, the PEO350 sample exhibited a similar j_corr_ (0.014 mA/cm^2^) and a more noble E_corr_ value of −0.94 V, suggesting improved corrosion resistance and delayed onset of degradation. Similarly, Vida et al. [30] reported j_corr_ values in the range of 0.00073 to 0.002 mA/cm^2^ for as-cast Zn-Mg alloys. Although these are slightly lower than the values obtained in our study for the untreated alloy, the PEO-treated samples, particularly ZnMg PEO350, show a better corrosion performance. Moreover, our impedance results for PEO350 reveal a significantly higher charge transfer resistance and larger Nyquist semicircle compared to the untreated alloy and other PEO variants. This supports the enhanced corrosion protection performance of the dual-layered PEO oxide film, which provides a dense and stable barrier not observed in the as-cast alloys studied previously.

Prior to salt solution exposure, the surface of ZnMg-PEO250 displayed a relatively smooth morphology, as illustrated in Figure 1a, exhibiting fine and evenly distributed porosity. However, following the corrosion test, the surface underwent visible changes, characterised by the emergence of discrete traces of fine cracks, the pores became more irregular and deep in some regions, and the overall texture underwent slight degradation, as depicted in Figure 5a. Despite the oxide layer maintaining its adhesion and integrity, these alterations mark the onset of a localised deterioration process due to extended exposure to the 0.9% NaCl solution.

As illustrated in Figure 1b, the surface of the ZnMg-PEO300 sample exhibited a relatively rough texture, characterised by irregular, medium-sized pores prior to the electro-chemical corrosion resistance test. However, the surface underwent significant alterations following the test, as illustrated in Figure 5b. The alterations included the development of deeper pores, “open” edges, and a fragmented texture, accompanied by the presence of evident cracks between the oxidic regions. Additionally, exposed areas, potentially indicating a visible metallic substrate, were identified. Despite the fact that the coating was initially found to be relatively continuous and uniform, significant degradation was observed after the corrosion test, with indications of local delamination and penetration of the corrosive medium.

In the case of ZnMg-PEO350, the surface before corrosion was found to be dense (Figure 1c). The porosity appeared fine and uniformly distributed, with round and small pores, suggesting a controlled PEO process. No obvious cracks were evident, and the texture was compact and homogeneous. After the corrosion resistance test, the surface retained a generally stable appearance with minor changes (Figure 5c). The morphology of the ZnMg-PEO350 sample exhibited remarkable robustness, maintaining its structural integrity even under exposure to a saline environment. This observation is further substantiated by the presence of a well-formed oxide structure, which demonstrated notable resistance to the effects of ionic attack. The presence of slightly pronounced pores is observed in some regions, but no major cracks have been identified and no aggressive exfoliation or openings in the oxide layer were observed [31].

As illustrated in Figure 1d, the surface of sample ZnMg-PEO400 appears to be quite rough, characterised by the presence of large, deep, and irregularly distributed pores. The structure appears to be more severely formed, exhibiting traces of microcracks within oxidised regions. The layer density was found to be more uneven in comparison to ZnMg-PEO350. Following the corrosion process (see Figure 5d), the surface of the material underwent significant alterations. The pores became more open, the microcracks became more pronounced, and local delamination or exfoliation was observed in some regions. The overall texture indicated a compromise in the integrity of the oxide layer. At a voltage of 400 V, the oxide layer that is initially formed is characterised by increased thickness. However, this layer is susceptible to cracking and mechanical instability [32]. These vulnerabilities are further accentuated during the process of corrosion.

The substantial decline in the percentage of oxygen in ZnMg-PEO300 indicates a severe degradation of the oxide layer in a saline environment. The analysis of sample PEO3 revealed the presence of a consistent oxide layer, a property that is known to contribute to enhanced corrosion resistance. Elemental sodium (Na) exhibits redox mobility, which suggests the potential for migration or partial removal from the surface during testing procedures.

The reduced content of ZnMg-PEO350 is indicative of a more stable structure, exhibiting reduced permeability to ion exchange. It has been observed that Mg exhibits increased reactivity in saline environments; in ZnMg-PEO400, a decline is noted, which is likely attributable to preferential dissolution. ZnMg-PEO250-PEO3 have been shown to retain Mg more effectively in the oxide layer, suggesting that they may facilitate more efficient initial oxidation. The almost zero value in ZnMg-PEO300 indicates a deep degradation of the phosphate layer. ZnMg-PEO350 retains the most P, indicating a stable and protective layer with corrosion inhibitory potential. Cl is a direct indicator of salt solution penetration. ZnMg-PEO400 has the highest accumulation, which is indicative of a porous or cracked layer. Furthermore, ZnMg-PEO250 and ZnMg-PEO350 have been shown to offer effective protection against Cl^−^ penetration. ZnMg-PEO350 appears to retain the greatest quantity of K, a phenomenon that may be attributable to a denser oxide layer and a propensity to incorporate cationic species. The elevated ZnMg-PEO300 content is indicative of substrate exposure, manifesting as damaged or thin oxide layers. ZnMg-PEO350 and ZnMg-PEO400 both exhibited moderate values, indicative of an effective oxidative barrier [33].

In comparison with the chemical compositions that have been determined on oxidised surfaces following PEO, the following observations were made: A decline in oxygen (O) was observed in ZnMg-PEO250 (–1.3%) and ZnMg-PEO400 (–1.3%), while ZnMg-PEO350 (0.2%) exhibited a marginal increase. Conversely, a substantial decrease was noted in ZnMg-PEO300 (–11.1%). The observed decrease in oxygen levels is indicative of the degradation of the oxide layer that had been formed during the PEO treatment. ZnMg-PEO300 exhibited the most significant degradation of the protective layer. ZnMg-PEO350 appears to be the most stable, with the oxide layer preserved. Na is mobile on the surface, i.e., it is not anchored in the compounds, and can be washed or replaced during the corrosion test. The decline in Na levels in ZnMg-PEO350 indicates that the material is becoming more compact and less permeable. For ZnMg-PEO250 and ZnMg-PEO400 samples, Na could be concentrated by diffusion from the saline environment or local restructuring. For the percentages of Mg, slight increases were observed at ZnMg-PEO250-ZnMg-PEO350 (+0.4–0.1%), while a decrease was noted at ZnMg-PEO400 (−0.5%). This is attributable to the reactivity of Mg, which readily dissolves in a saline medium. In the case of ZnMg-PEO400, the loss of Mg can be attributed to selective dissolution. The stability of the other samples indicates satisfactory initial oxidation and, to a certain extent, effective protection. P has been shown to function as a protective element, playing an inhibitory role in a saline environment. The loss of this layer, particularly in ZnMg-PEO300, is indicative of a breakdown in the protective barrier. ZnMg-PEO350 maintains a compact, protective layer of phosphorus. K represents a mobile and weakly bound cation within the layer. The observed variability indicates that it does not directly impact corrosion resistance. The slight increase in ZnMg-PEO350 may be related to efficient retention in the layer. Zn is derived from the metallic substrate; an increase in Zn results in the erosion of the oxide layer. In the case of ZnMg-PEO300, the layer exhibited a pronounced response, enabling substrate detection. The increase observed for ZnMg-PEO350 is moderate, indicating a potential for localised exposure.

As shown in Table 5, the corrosion process initiates the degradation of the material. P continued to be maintained, although with a marginally more discontinuous distribution. Cl has been identified as a new phenomenon, indicating the presence of chloride ions from the salt solution that have penetrated the layer. Furthermore, Na and K have been redistributed, and there is a possibility that they have migrated due to exposure to the solution. A slight increase in intensity is observed for Zn, indicating localised erosion of the layer and partial exposure of the substrate. A slight decrease in Mg was observed, which may be indicative of preferential dissolution within a saline environment. Cl is an indicator of the penetrative action of the salt solution and the initiation of the chemical degradation process. In general, the layer retains its functionality; however, processes of ionic migration and localised attack begin, especially in the defect areas. The intensity of oxygen is slightly diminished, indicating that the oxide has been partially consumed or converted. The assertion that oxygen is “partially consumed or converted” signifies that oxygen, existing in the form of metal oxides (e.g., ZnO, MgO or oxygenated phosphates), is capable of undergoing specific processes when exposed to a saline solution (0.9% NaCl):

1. Either consumed by dissolution reactions: in the presence of Cl^−^ ions, metal oxides can be dissolved:
  ZnO + 2H^+^ → Zn^2+^ + H_2_O (1)
  MgO + 2H^+^ → Mg^2+^ + H_2_O (2)

The oxygen is thus “lost” as water and the metal is released as an ion.

2. Participates in the formation of new products: Oxygen can react with chlorides or corrosion intermediates, resulting in:
  - Hydroxides (less protective):
    ZnO + H_2_O → Zn(OH)_2_ (3)
  - Complex chlorinated oxihalides or chlorinated zinc species:
    ZnO + 2Cl^−^ + 2H^+^ → ZnCl_2_ + H_2_O (4)
3. Local cathodic reduction loss: in anodic or micro-cracked areas:
  O_2_ + 4e^−^ + 4H^+^ → 2H_2_O (5)

**Figure 6:**
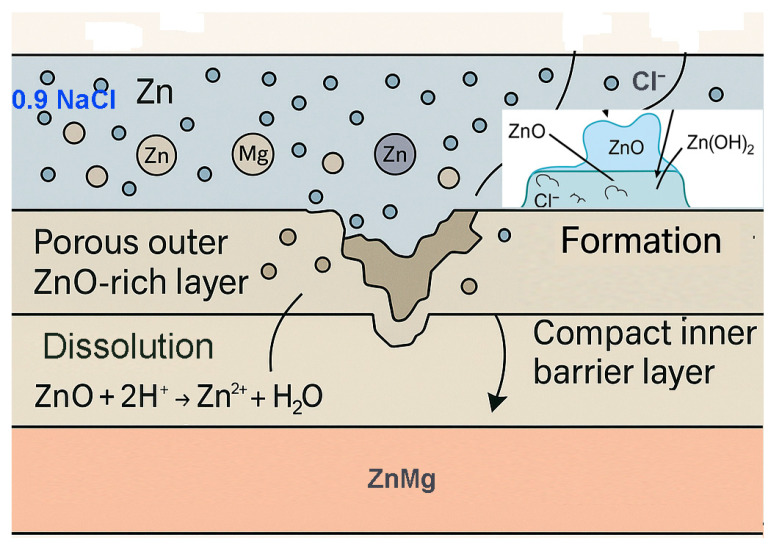
Schematic corrosion mechanism of PEO samples in 0.9NaCl.

**Figure 7:**
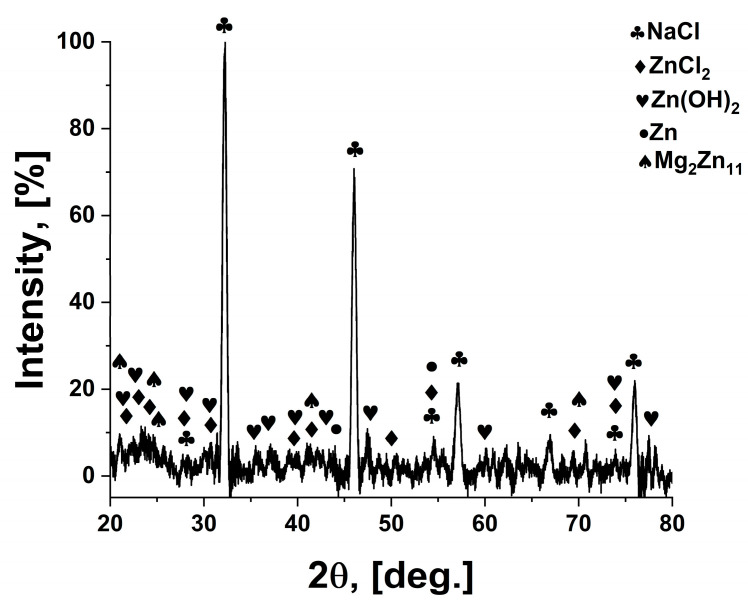
Diffractogram of ZnMg-350PEO sample subjected to corrosion in 0.9%NaCl.

This reaction consumes dissolved oxygen and has the potential to destabilise the local chemistry of the oxide layer. The conversion of oxygen on the surface from stable forms (oxides) to other forms is a process of significant scientific interest. The presence of soluble metal ions (e.g., Zn^2+^, Mg^2+^) and by-products (e.g., hydroxides, chlorides) or water is indicative of redox reactions, Figure 6 and confirmed by XRD analysis in Figure 7.

After corrosion of the oxidised layer, based on XRD analysis, Figure 7, the following compounds were identified: NaCl (halite), Zn(OH)_2_, ZnCl_2_, Zn, Mg_2_Zn_11_. The identification of the compounds on the surface confirms the results of the EDS analysis and the chemical reactions taking place between the metallic material and the etching solution. A decrease in the intensity of the characteristic peaks for Zn and Mg_2_Zn_11_ is observed. The presence of natrium chloride is due to the deposition of this compound, present in the electrolyte solution, in the pores on the surface. Phase identification and peak indexing were performed using Match! version 3.16 software, in conjunction with the Crystallography Open Database (COD). The following reference CIF files were used to ensure accurate phase assignment: Zn—COD: 96-900-8523, ZnO—COD: 96-900-4182, Mg_2_Zn_11_—COD: 96-152-2822, ZnCl_2_—COD: 96-152-8213, Zn(OH)_2_—COD: 96-152-7884.

The decline in the oxygen signal, as observed through EDS analysis, is indicative of these transformations and the degradation of the protective layer. For sample ZnMg-PEO300, as illustrated in the analysis of element distribution prior to corrosion (Supplementary File Figure S1b), it was observed that O exhibited a weak and non-uniform distribution, indicative of an incomplete or porous oxide layer. Zn manifested a high intensity, suggesting either partial substrate exposure or a thin oxide layer. The presence of Na and P elements was marginal, indicating reduced phosphate incorporation. Mg and K elements were also weakly visible, consistent with a weakly oxidised composition. Following the corrosion process, the presence of Cl becomes visible in certain areas, exhibiting both intense and diffuse characteristics. This phenomenon can be attributed to the substantial penetration of ions from the saline solution. Concurrently, the presence of oxygen was found to be minimal, suggesting significant dissolution of the oxide. The intensity of Zn exhibited a pronounced response, indicating extensive exposure of the substrate. In contrast, the signals from Na and P indicated a marked weakness, possibly attributable to their complete dissolution, resulting in the dissolution of phosphates and sodium. The signals from Mg and K were either weak or absent, suggesting preferential dissolution of these elements. The oxide layer composition has been severely compromised. The presence of chlorine, both in terms of quantity and intensity, along with the amplification of the zinc signal, serves to substantiate the degradation of the protective layer and the subsequent exposure of the metallic substrate.

An analysis of the distribution of elements on the surface of the ZnMg-PEO350 sample, as illustrated in the Supplementary File (see Figure S1c [before]), reveals a robust and homogeneously distributed presence of O before corrosion, thereby confirming the formation of an oxide layer. In contrast, P exhibits a clear distribution. Evidence suggests that the presence of phosphates within the layer is indicative of their integration. The uniform distribution of Na and K elements is attributable to the influence of the electrolyte (Na_3_PO_4_ + KOH). The moderate signal observed for Zn can be ascribed to the effective coating of the substrate, while the slight visibility of Mg is attributed to its incorporation within the oxide structure. As shown in Table 5, post-corrosion analysis reveals the retention of elements O and P in terms of intensity and distribution. This is attributable to the layer’s effective resistance to corrosion. In contrast, the presence of element Cl appears diminished and localised, attributed to a reduced penetration of corrosive ions. The presence of Zn is slightly accentuated in certain areas, yet it does not dominate, as the oxide layer continues to provide protection to the substrate. Additionally, Na and K undergo slight redistribution or concentration at the surface, an outcome of their interaction with the saline environment. The elemental distributions confirm a superior chemical resistance of this sample compared to the two previously analysed. Furthermore, the Cl^−^ ions do not penetrate deeply, and the oxide layer components are maintained.

As demonstrated in Figure S1d (before) and Table 5 (after), the distribution of elements was found to be adequate; however, it was slightly non-uniform. This observation indicates efficient oxidation of oxygen prior to corrosion, though with variable density. The presence of P is observed in the layer; however, its homogeneity is found to be inferior to that of ZnMg-PEO350. The elements Na and K are discernible and originate from the PEO electrolyte solution, exhibiting a distribution that encompasses the entire surface. The intensity of Zn is notable due to the imperfect coverage of the substrate by the layer. Following the oxidation process, the elemental magnesium is represented only in a minor way, given that it underwent only marginal oxidation. Following the corrosion process, the presence of Cl is detected, exhibiting an intense, diffuse distribution, attributed to the high penetrability of the layer. A distinct decrease in intensity corresponding to elements O and P was observed, attributable to the impact on the oxide and phosphate layers. Concurrently, Zn becomes predominant due to the exposure of the substrate in multiple regions. The redistribution of Na and K elements, potentially resulting from migration or dilution due to salt attack, is a notable phenomenon. The oxide layer exhibited significant deterioration under the influence of the saline environment. The penetration of Cl ions is extensive, which is conducive to the dissolution of oxide components and the exposure of the metallic substrate.

Subsequent to the corrosion tests, the PEO samples exhibited divergent behaviours, which were found to be directly influenced by the structure and quality of the oxide layer initially formed at varying voltages. The ZnMg-PEO250 sample, which was subjected to a voltage of 250 V, exhibited a relatively stable surface following the corrosion process. This was characterised by minimal layer degradation and moderate Cl^−^ ion penetration, which collectively suggested a satisfactory level of protection. However, it should be noted that the efficacy of this protection was constrained by the limited thickness of the layer. The ZnMg-PEO300 sample, oxidised at 300 V, exhibited the most significant damage: SEM images illustrate substantial cracking and delamination of the layer, while the element distribution demonstrates substantial chlorine ion penetration and significant oxygen and phosphorus losses. These observations are indicative of advanced corrosion and an initially unstable layer. In contrast, PEO3, treated at 350 V, demonstrated optimal performance. The surface remained compact, with no visible cracks, and EDS maps after corrosion showed that oxygen, phosphorus and chlorine, in minimal quantities, had penetrated the oxide layer, indicating that it was dense, homogeneous and protective. The ZnMg-PEO400 sample, obtained at 400 V, exhibited an initial layer of considerable thickness, yet demonstrated a propensity towards exfoliation and substantial penetration of corrosive ions. This observation suggests that the high voltage may have induced a brittle layer, which is susceptible to cracking and structural instability.

The main steps of the 0.9% NaCl saline corrosion model of oxidised layers are as follows:

- The penetration of Cl^−^ ions through the pores of the PEO layer (especially in defective or cracked areas);
- Chemical attack on metal oxides: Cl^−^ destabilises Zn and Mg oxides by dissolution reactions;
- Dissolution of the phosphate phases, especially in ZnMg-PEO300, by replacing PO_4_^3−^ anions by Cl^−^;
- Exposure of the metallic substrate ZnMg, which starts to oxidise galvanically in the presence of water and dissolved oxygen;
- Corrosion products formation (hydroxides, chlorides, oxyhalogens).

## 5. Conclusions

Among the investigated conditions, the sample ZnMg-PEO350 (treated at 350 V) exhibited the most favourable morphology and electrochemical performance, due to an optimal balance between oxide growth and structural integrity. At this voltage, the oxide layer showed a compact and stable structure, with high oxygen, phosphorus and magnesium content, and minimal zinc signal from the substrate, indicating effective coverage. Voltage 350 V has been determined to be the optimal setting, resulting in a layer that is abundant in O, P, Mg and contains minimal Zn (from the substrate), suggesting effective coverage and efficient oxidation. In the context of electroplating, it has been demonstrated that low voltages (250 V) can result in the formation of thin, Zn-rich layers characterised by shallow oxidation. It is noteworthy that very high voltages (400 V) have the potential to induce defects, such as cracks and uncontrolled discharges. These defects can compromise the uniformity of the layers and the effective incorporation of elements during the process. ZnMg-PEO300 has been identified as the primary affected component, exhibiting oxide losses, elevated Cl levels, and the formation of an unstable layer. ZnMg-PEO350 exhibited the optimal performance, characterised by its stable oxide composition, minimal infiltration, and effective coating properties. ZnMg-PEO250 and ZnMg-PEO400 were found to be intermediate types, with ZnMg-PEO250 being more stable than ZnMg-PEO400, which allowed chloride ingress.

ZnMg-PEO350 demonstrated the optimal performance in terms of stability within a corrosive environment. The results of this study provide compelling evidence that the layer formed at 350 V was both dense and adherent, and exhibited a high level of resistance to Cl^−^.

When subjected to identical testing conditions, the corrosion behaviour of the PEO samples was predominantly influenced by the quality of the obtained oxide layer. The ZnMg-PEO350 sample, which was treated at 350 V, offered the optimum protection, due to the optimal balance between thickness, porosity and chemical stability. As can be seen from the data presented, ZnMg-PEO250 provided adequate protection, though this was limited to a certain degree. By contrast, ZnMg-PEO300 was found to be the most vulnerable of the samples, whilst ZnMg-PEO400, despite its thickness, proved unstable in corrosive environments due to its brittle structure.

## Figures and Tables

**Table 1:** Plasma electrolyte oxidation parameters.

Sample	U Initial—Passivation (t Initial 30 s)	U Final (t Final 150 s)	Frequency	Filling Factor
ZnMg-PEO250	150 V	250 V	1 kHz	10%
ZnMg-PEO300	150 V	300 V	1 kHz	10%
ZnMg-PEO350	150 V	350 V	1 kHz	10%
ZnMg-PEO400	150 V	400 V	1 kHz	10%

**Table 2:** Surface chemical composition of ZnMg-PEO250-ZnMg-PEO400 samples.

Element	ZnMg- PEO250	ZnMg- PEO300	ZnMg- PEO350	ZnMg- PEO400	Error (Avg)
	**at.%**	**at.%**	**at.%**	**at.%**	**at.%**
O	48.7	45.6	52.0	48.6	0.38
Na	14.5	15.6	15.4	16.8	2.45
Mg	2.8	3.9	3.9	2.3	0.13
P	9.7	5.7	9.7	10.2	0.10
K	0.5	0.3	0.7	0.2	0.01
Zn	23.8	28.9	18.3	21.9	0.25

**Table 3:** Electrochemical parameters determined from Tafel diagrams for PEO-treated ZnMg samples oxidised.

System	Corrosion Process Parameters						
	E(I = 0) (mV)	j_corr_ (mA/cm^2^)	v_corr_ (mm/yr)	Rp ohm.cm^2^	β_a_ (mV/dec)	−β_c_ (mV/dec)	OCP (mV)
Initial	−1067	0.277	3.87	112	184	173	−1059
ZnMg-PEO250	−967	0.364	4.9	80	166	128	−970
ZnMg-PEO300	−1026	0.041	0.587	619	109	179	−1006
ZnMg-PEO350	−979	0.014	0.199	2210	217	321	−940
ZnMg-PEO400	−988	0.032	0.451	1180	212	223	−950

**Table 4:** Electrical parameters obtained by fitting EIS data using the equivalent circuit R(Q(R(QR))) for the untreated and PEO-treated ZnMg alloy (ZnMg-PEO250-ZnMg-PEO400) in 0.9% NaCl solution.

Sample	R_s_ Ohm.cm^2^	CPE1		R_1_ Ohm.cm^2^	CPE2		R_2_ Ohm.cm^2^	
		Q1 Ss^n^/cm^2^	n1		Q2 Ss^n^/cm^2^	n2		P.E. %
Initial	22	2.2·10^−6^	0.9	20	2.2·10^−4^	0.56	232	
ZnMg-PEO250	35	1.51·10^−7^	0.81	71	4.8·10^−4^	0.51	90	71.83
ZnMg-PEO300	43	2.1 10^−4^	0.38	309	4.8·10^−4^	0.36	296	93.53
ZnMg-PEO350	83	4.11·10^−7^	0.7	1471	4.66·10^−7^	0.72	1962	98.64
ZnMg-PEO400	18	6.69·10^−5^	0.67	419	5.5·10^−5^	0.86	425	95.23

**Table 5:** Surface chemical composition after electro-chemical corrosion resistance tests.

Element	At.% ZnMg-PEO250	At.% ZnMg-PEO300	At.% ZnMg-PEO350	At.% ZnMg-PEO400	At.% Error (Avg)
O	46.5	29.0	53.9	47.0	0.28
Na	16.4	17.6	9.6	16.8	1.48
Mg	3.2	5.1	3.7	1.7	0.1
P	9.6	0.3	10.1	8.5	0.08
Cl	0.3	1.7	0.6	3.2	0.0
K	0.3	0.1	0.8	0.3	0.0
Zn	23.7	46.2	21.3	22.5	0.13

## Data Availability

The original contributions presented in this study are included in the article. Further inquiries can be directed to the corresponding author.

## Supplementary Materials

The following supporting information can be downloaded at: https://www.mdpi.com/article/10.3390/ma18174064/s1, Figure S1: Mapping of the main elements on the surface after PEO; Figure S2: Sectional SEM of PEO samples; Figure S3: Line scan of cross section of PEO coatings.
