# Supplementary material for: PEO Treatment for Improved Corrosion Resistance in a Zn-Mg Alloy: Electrochemical and Structural Analysis
Source: Materials (Basel). 2025 Aug 29;18(17):4064. doi: 10.3390/ma18174064 (PMC12429804; doi:10.3390/ma18174064)
Supplement: Supplementary file 1 [file materials-18-04064-s001.zip › materials-3804709-supplementary.pdf]

Supplementary file of Paper: PEO Treatment for Improved Corrosion Resistance in a Zn-Mg Alloy: Electrochemical and Structural Analysis

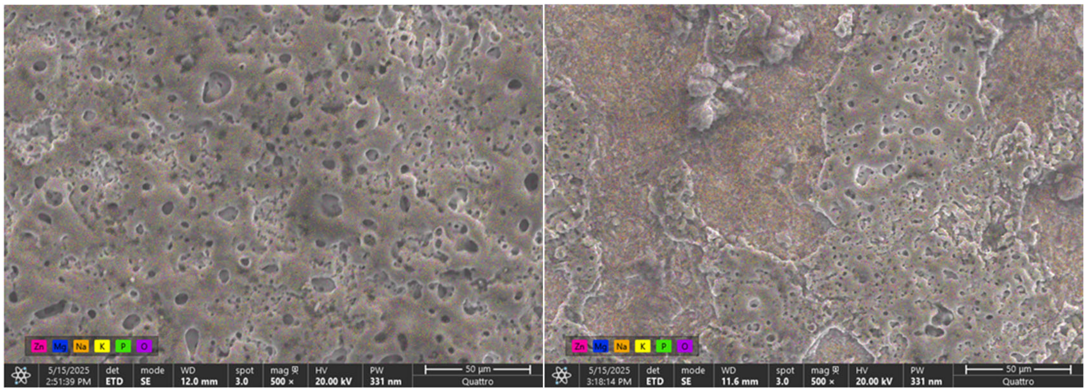

a b

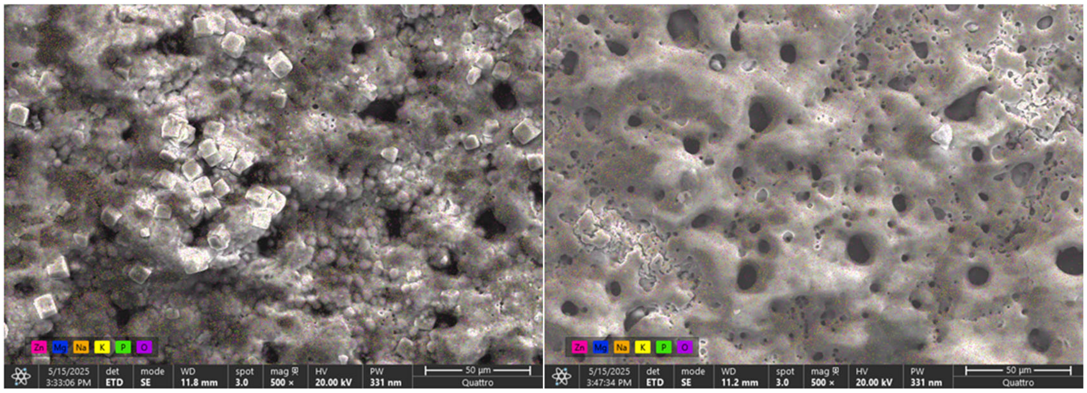

c d

Figure S1 Mapping of the main elements on the surface after PEO

Oxidized layer thicknesses >

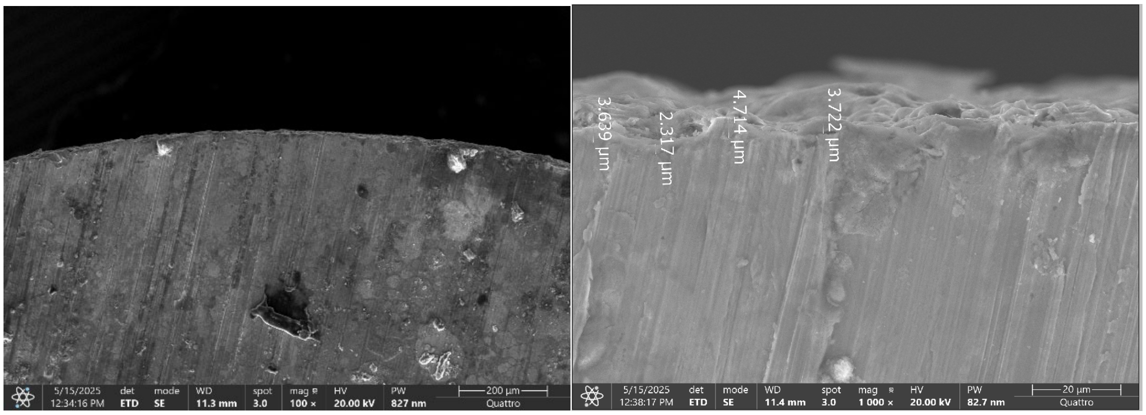

ZnMg-PEO250

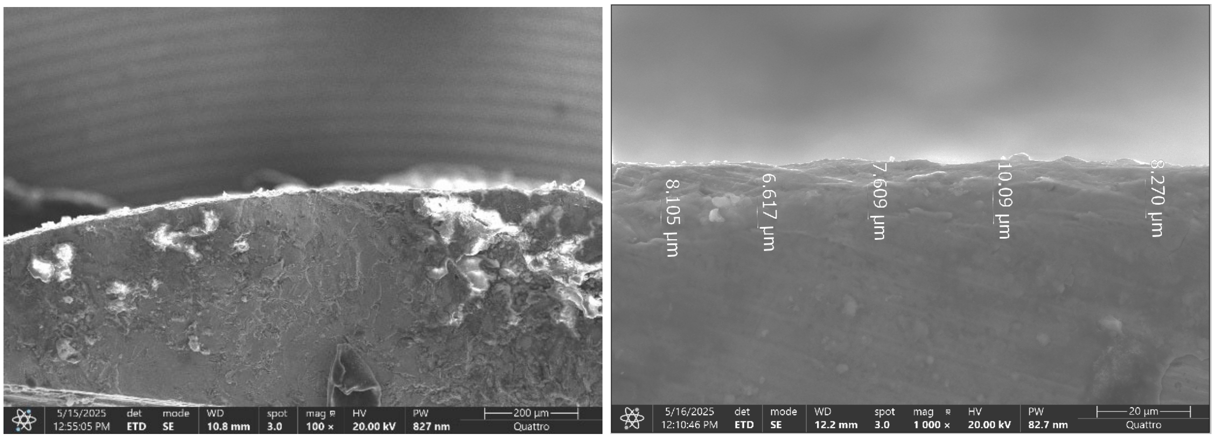

ZnMg-PEO300

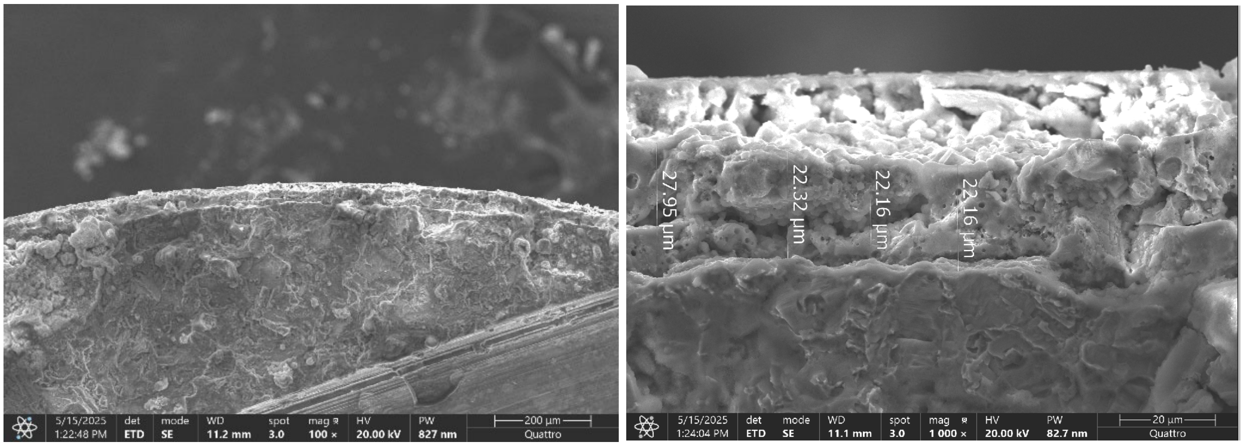

ZnMg-PEO350

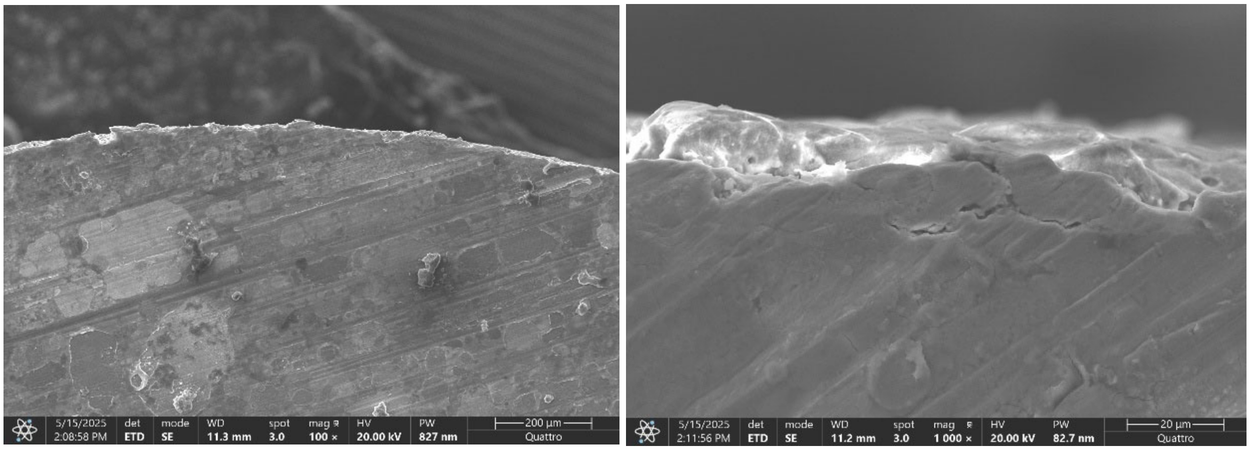

ZnMg-PEO400

Figure S2. Sectional SEM of PEO samples

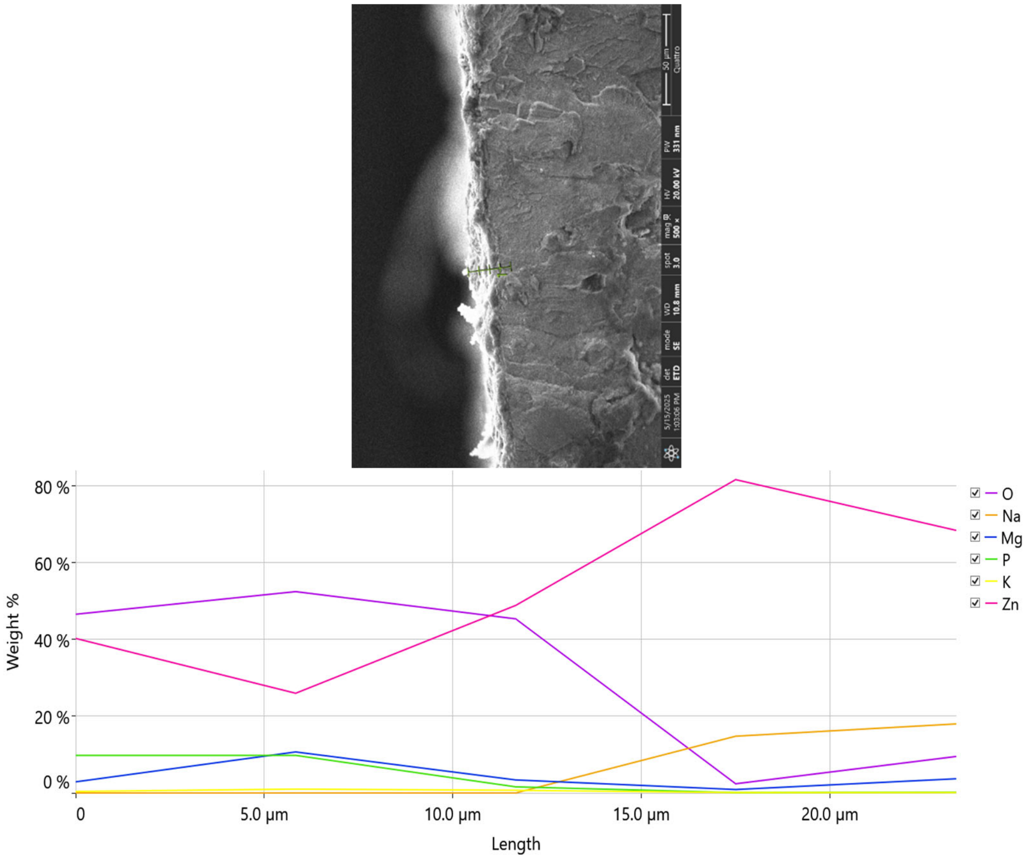

Figure S3. Line scan of cross section of PEO coatings
